# Podocyte Developmental Defects Caused by Adriamycin in Zebrafish Embryos and Larvae: A Novel Model of Glomerular Damage

**DOI:** 10.1371/journal.pone.0098131

**Published:** 2014-05-20

**Authors:** Cristina Zennaro, Massimo Mariotti, Michele Carraro, Sara Pasqualetti, Alessandro Corbelli, Silvia Armelloni, Min Li, Masami Ikehata, Milan Clai, Mary Artero, Piergiorgio Messa, Giuliano Boscutti, Maria Pia Rastaldi

**Affiliations:** 1 Department of Medical, Surgery and Health Sciences, Università degli Studi di Trieste, Trieste, Italy; 2 Renal Research Laboratory, Fondazione IRCCS Ca' Granda Ospedale Maggiore Policlinico & Fondazione D'Amico per la Ricerca sulle Malattie Renali, Milano, Italy; 3 Department of Biomedical, Surgical and Dental Sciences, University of Milano & IRCCS Orthopedic Institute, Milano, Italy; 4 Gruppo Ospedaliero San Donato Foundation, Milano, Italy; 5 Azienda Ospedaliero-Universitaria Ospedali Riuniti di Trieste, Trieste, Italy; 6 Department of Cardiovascular Research- Unit of Bio-imaging, Mario Negri Institute for Pharmacological Research, Milano, Italy; 7 Division of Nephrology, Dialysis, and Renal Transplant, Fondazione IRCCS Ca' Granda Ospedale Maggiore Policlinico, Milano, Italy; Biomedical Research Foundation of the Academy of Athens, Greece

## Abstract

The zebrafish pronephros is gaining popularity in the nephrology community, because embryos are easy to cultivate in multiwell plates, allowing large number of experiments to be conducted in an *in vivo* model. In a few days, glomeruli reach complete development, with a structure that is similar to that of the mammalian counterpart, showing a fenestrated endothelium and a basement membrane covered by the multiple ramifications of mature podocytes. As a further advantage, zebrafish embryos are permeable to low molecular compounds, and this explains their extensive use in drug efficacy and toxicity experiments. Here we show that low concentrations of adriamycin (i.e. 10 and 20 µM), when dissolved in the medium of zebrafish embryos at 9 hours post-fertilization and removed after 48 hours (57 hpf), alter the development of podocytes with subsequent functional impairment, demonstrated by onset of pericardial edema and reduction of expression of the podocyte proteins nephrin and wt1. Podocyte damage is morphologically confirmed by electron microscopy and functionally supported by increased clearance of microinjected 70 kDa fluorescent dextran. Importantly, besides pericardial edema and glomerular damage, which persist and worsen after adriamycin removal from the medium, larvae exposed to adriamycin 10 and 20 µM do not show any myocardiocyte alterations nor vascular changes. The only extra-renal effect is a transient delay of cartilage formation that rapidly recovers once adriamycin is removed. In summary, this low dose adriamycin model can be applied to analyze podocyte developmental defects, such as those observed in congenital nephrotic syndrome, and can be taken in consideration for pharmacological studies of severe early podocyte injury.

## Introduction

Damage to the podocyte, the highly ramified cell that surrounds and completely covers with primary and secondary processes the basement membrane of the glomerular capillary, accounts for the majority of proteinuric diseases of the kidney, and is prominent in nephrotic syndrome due to primary or secondary causes [1]. In these diseases podocytes “flatten”, losing the ordered arrangement of primary and secondary processes separated by the slit diaphragm, because of a profound alteration of the actin cytoskeleton [2].

Despite novel insights gained in the last decade on specific molecules and pathways involved in the pathogenesis of proteinuric diseases, there is still the need of more complete understanding of the events leading to podocyte damage and of models to be utilized for drug screening to translate research results into valuable therapies.

In this framework, the zebrafish (*Danio rerio*) pronephros constitutes a simple and accessible system to study *in vivo* numerous aspects of renal cell biology. The transparency of zebrafish larvae and the 48 h time-frame of complete pronephros maturation makes it feasible to analyze glomerular and tubular development and study numerous aspects of cell differentiation in an *in vivo* setting [3].

These aspects become particularly important in consideration of the accessibility of the glomerular structure and its similarity to the mammalian glomerulus, with a capillary composed by a fenestrated endothelium lying on a basement membrane, externally enwrapped by the highly ordered web formed by primary and secondary podocyte processes [4].

Expression studies, the use of morpholino knockouts, and more advanced transgenic techniques allowing inducible podocyte injury [5] have demonstrated that several molecules relevant for development and maintenance of podocyte structure in mammals, such as nephrin, podocin, and Wilms tumor 1 (wt1), are indeed expressed in the zebrafish glomerulus and conserve similar function [6], [7]. Another important advantage of zebrafish models is the possibility to rapidly assess glomerular injury by the appearance of pericardial edema, due to loss of proteins through the glomerular filter [8].

In rodents, nephrotic syndrome can be induced by injection of several compounds with different effects; protamine sulfate leads to rapidly reversible podocyte alterations similar to human minimal change disease [9]. Depending on the cumulative dosage, puromycin aminonucleoside causes either a mild and reversible glomerular damage resembling minimal change disease or more severe lesions evolving towards the development of glomerulosclerosis, thereby mimicking human focal segmental glomerulosclerosis (FSGS) [10]. A single injection of adriamycin, a small molecule (579.98 dalton) which accumulates mainly in the kidney, is capable of causing a severe form of FSGS in rats and Balb/c mice [11].

It is noteworthy that puromycin aminonucleoside microinjection was found capable of causing podocyte damage also in the zebrafish [8], although requiring much higher dosages than those used *in vitro* and in rodent models. The effects of adriamycin on fish pronephros, however, have not been explored.

Zebrafish embryos and early larvae absorb low molecular weight substances through skin and gills up to seven days of life. This property has been exploited in various experimental settings, because dose-dependent and time-dependent effects on organ and tissue development can be rapidly assessed by simply dissolving the compound of interest in the medium [12].

Therefore, we designed the present study to determine if early exposure to adriamycin could generate a model of podocyte developmental damage that would be easy to reproduce and evaluate.

## Materials and Methods

### Embryo collection

Zebrafish were raised and maintained as previously described [13]. Embryos were generated by natural pair-wise mating and were kept and handled for all experiments in E3 medium (5 mM NaCl, 0.17 mM KCl, 0.33 mM CaCl_2_, 0.33 mM MgSO_4_).

### Ethics statement

All experimentations in the zebrafish Lab (IRCCS R. Galeazzi, Milan, Italy) conform to the ITA and EU guidelines on research practice (European directory 2007/526/CE). The zebrafish experimentation and protocols were approved by the Italian Ministry of Health and ASL Varese (Prot. N. 014AVB0020033). When appropriate, anesthesia was induced by 1∶20 to 1∶100 dilution of 4 mg/ml Tricaine (Sigma-Aldrich, Milan, Italy) and live fish were positioned in methylcellulose for microscope observation.

For the Palb test a committee of Italian Health Ministry approved the experimental protocol (Prot. N. 1120 - August, 7, 2102) in compliance with Italian regulation (D.L.vo 116/92).

### Glomerular permeability to albumin test (Palb)

The Palb test was used to preliminarily assess which concentration of adriamycin could actively modify the permeability of isolated rat glomeruli. The technique is extensively in use in our laboratory [14], [15]. Briefly, glomeruli were isolated from 3 month old Sprague Dawley rats using standard sieving techniques in medium containing 5 g/dL of bovine serum albumin. Each of 15 to 20 glomeruli per animal were videotaped through an inverted microscope before and after a medium exchange to one containing 10 g/L of bovine serum albumin. The medium exchange created an oncotic gradient across the basement membrane, resulting in a glomerular volume change [ΔV = (Vfinal−Vinitial)/Vinitial], which was measured offline using a video-based image analysis program (SigmaScan Pro; Jandel Scientific Software, Erkrath, Germany). The computer program determines the average radius of the glomerulus in two-dimensional space, and the volume is derived from the formula V = 4/3πr3. The magnitude of ΔV was related to the albumin reflection coefficient (σalb) by the following equation: (σalb) experimental  = (ΔV)experimental/(ΔV) control; the σalb of control glomeruli is assumed to be equal to 1. The Palb is defined as (1−σalb) and describes the movement of albumin subsequent to water flux. When σalb is 0, albumin moves across the membrane with the same velocity as water, and Palb is 1.0; conversely, when σalb is 1.0, albumin cannot cross the membrane with water, and Palb is 0. Palb values higher than the cut-off value of 0,5 are considered positive, indicating an increase of permeability through the glomerular barrier.

### Embryo exposure to adriamycin

Dose-dependent and time-dependent studies were performed by adding 5 to 40 µM adriamycin to the E3 medium of 3 to 10 hours post fertilization (hpf) old zebrafish embryos. The substance was left in the medium for 48 h, then animals were washed several times with E3 medium and were immersed in medium without adriamycin until the end of the experiments. A total number of 340 animals were used in these studies.

### Measurement of body length and pericardial edema

Body length was determined using digital photographs and applying the length measurement tool of Sigma Scan Pro software (Jandel Scientific Softaware).

Pericardial edema was estimated using the area measurement tool of the same software to assess the cross-sectional pericardial area and the total body area. Results, obtained from 45 adriamycin-treated embryos and normalized to those obtained from 45 untreated controls, were expressed as cardiac/body ratio.

All pictures were taken at the same resolution and magnification with the live fish positioned in a lateral orientation.

### Mannitol exposure

Freshwater fish, such as *D. rerio*, are constantly surrounded by very low osmolar medium. Therefore, the ability to exclude water is essential to survival and depends on gills, kidney, and skin, which all contribute to maintain proper internal volume and osmolarity. In consideration of this behavior, formation of edema could be due either to alteration of kidney function and/or to malfunction of other water barriers [16]. To determine if appearance of pericardial edema could result from a direct effect of adriamycin on glomerular development and exclude alteration of the other water barriers, the osmotic force that drives the fish was removed by addition of mannitol, as described [16]. To this end, 175 mM mannitol was added to 72 hpf old adriamycin-treated and control early larvae (45 animals/group), and images were taken at 0, 5, 20 and 54 hours after mannitol addition.

### RNA Isolation and QRT-PCR

Total RNA was extracted from 60 early larvae exposed to adriamycin and 60 controls at 72 and 120 hpf, using Trizol Reagent (Invitrogen, Life Technologies, Milan, Italy), and RNA concentration was determined by Nanoquant (Tecan, Italy). One microgram of total RNA was reverse transcribed using MMLV reverse transcriptase (Applied Biosystems). The following oligonucleotides were applied as forward and reverse primers for quantitative Real Time RT-PCR (QRT-PCR): *Danio rerio* nephrin 5′-CAG TCA CAG GCC TTA ACC CTT CAA-3′ (forward) and 5′-CGA GGC GTT GAT AAG CTC TCT GCT-3′(reverse); *Danio rerio* wt1a 5′-TGC TGA TCC TCC TTC TAG CC-3′ (forward) and 5′-GAA CGG AGG AGT GTG TTG TG-3′ (reverse); *Danio rerio* wt1b 5′-AGC CAA CCA AGG ATG TTC AG-3′ (forward) and 5′-GAA TGC CAT TAA AGT AGT TCC TC-3′ (reverse) and *Danio rerio* β-actin (house-keeping gene) 5′-CAG CAA GCA GGA GTA CGA TGA GT-3′ (foward) and 5′-TTG AAT CTC ATT GCT AGG CCA TT-3′ (reverse).

### Histology and immunostaining

For light microscopy, 40 embryos per group and time point were fixed in 4% buffered paraformaldehyde, dehydrated, and paraffin-embedded. 2–3 µm thick sections were cut by Microm HM450 sliding microtome and stained with hematoxylin eosin.

For transmission electron microscopy, conducted on 20 animals per group and time point, fixation was performed in a mixture of paraformaldehyde, glutaraldehyde, and phosphate buffer, and samples were embedded in resin.

For whole mount actin staining, 20 embryos per group and time point were fixed overnight at 4°C in 4% phosphate-buffered paraformaldehyde, then permeabilized at 4°C in PBS containing 2% Triton-X100, and incubated in FITC-phalloidin (Sigma) for 3 hours at room temperature.

### Glomerular function

A qualitative assay was employed to investigate glomerular blood filtration, as described by Hentschel et al. [8]. Briefly, a solution of FITC-labeled 70 kDa-dextran (Sigma) was injected into the cardiac venous sinus of 80 hpf old early larvae. 30 animals per group and time point were studied. Sequence images of live fish were generated using a Leica inverted microscope connected to a Leica DFC490 camera. The maximum fluorescence intensity of greyscale images of the cardiac area and the eye were measured using Sigma Scan Pro software and reported in relative units of brightness. For heart fluorescence measurements, the animals with significant amount of FITC-dextran trapped in the yolk sac were excluded from the analysis.

### Heart frequency measurement

The heartbeat frequency was determined by direct visual counting of ventricle beating using a stereomicroscope (Leica). Movies of the beating heart were recorded using an inverted microscope (Leica) connected to a videocamera, and capturing two full heart beats per second.

### Cartilage staining

For cartilage staining, 20 animals per group and time point were prepared by fixation in 4% phosphate-buffered paraformaldehyde and maintained in methanol at 20°C until use. Then, staining was performed by Alcian blue solution (1% hydrochloric acid, 70% ethanol and 0,1% Alcian blue).

### Vessel staining

At 72 hpf, 40 embryos per group were fixed in 4% paraformaldehyde for 2 hours at room temperature and stained for assessment of endogenous alkaline phosphate activity, as described [17].

### Statistical analysis

Data are presented as mean ± standard error. Comparison between groups of data was performed by one-way analysis of variance (ANOVA).

## Results

### Increase of Palb by adriamycin in rat glomeruli

**Figure 1:**
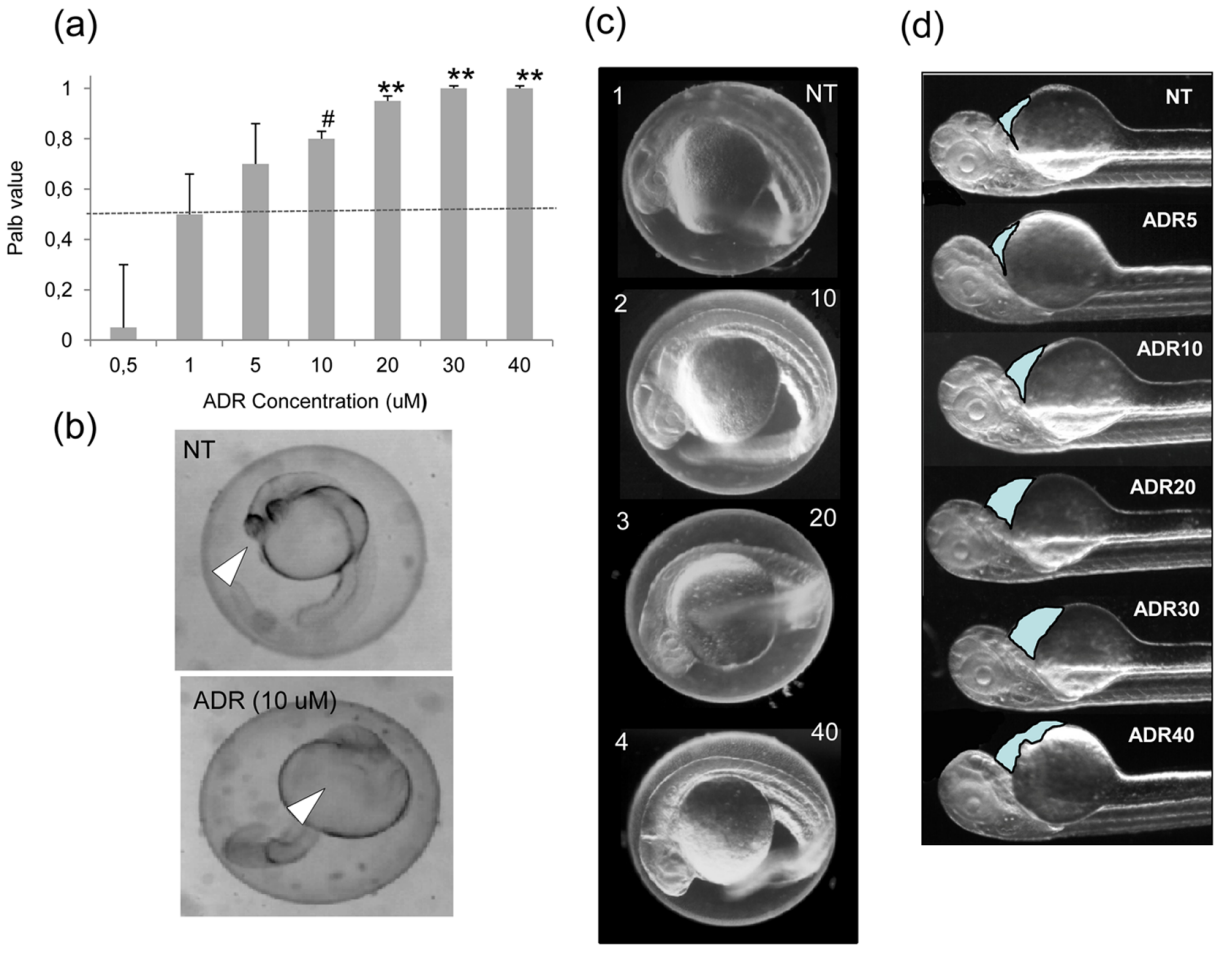
Adriamycin dose and time-dependent effects. (a) Palb test results showing dose-dependent effects of adriamycin on isolated rat glomeruli. The test is considered positive when Palb values are higher than 0.5 (dashed red line). Asterisks (**) indicate P<0.005 vs NT (NT =  Not Treated) and ^#^ P<0.05. (b) A representative example of the developmental defects caused by exposure to 10 µM adriamycin at 3 hpf. Images were taken at 24 hpf. As compared to a normally developing embryo (NT, left panel), the adriamycin-treated embryo (ADR) shows severe changes, such as the absence of eye development. Arrows point to the eye in NT embryo and its absence after ADR exposure. (c) Normal development of 24 hpf embryos exposed to 10–40 µM adriamycin (ADR) when ADR is dissolved in the medium at 9 hpf. Treated embryos (panels 2–4) are indistinguishable from the control (panel 1, NT). (d) At 48 hpf, as compared to the control (NT, top panel), fish exposed to adriamycin 10–40 µM start displaying evident dose-dependent pericardial edema (light blue areas). The effect is very mild at the adriamycin dosage of 5 µM (second panel from the top).

The Palb test demonstrated that adriamycin was able to increase permeability of isolated rat glomeruli starting from a concentration of 10 µM. The highest permeability values were obtained at concentrations of 20, 30 and 40 µM (Figure 1a).

### Adriamycin induces glomerular damage in zebrafish embryos

In order to establish the age of animals to be used in our experiments, 5 to 40 µM adriamycin was added to the E3 medium at different time points and washed out after 48 hours. By these experiments we observed that the earliest exposure (at 1–3 hpf) to any concentration of adriamycin caused severe developmental changes followed by death (Figure 1b).

Instead, when adriamycin was given at 9 hpf, embryos displayed normal development until hatching (Figure 1c). Noteworthy, the substance dose-dependently caused pericardial edema, which was evident at 72 hpf (Figure 1d).

**Figure 2:**
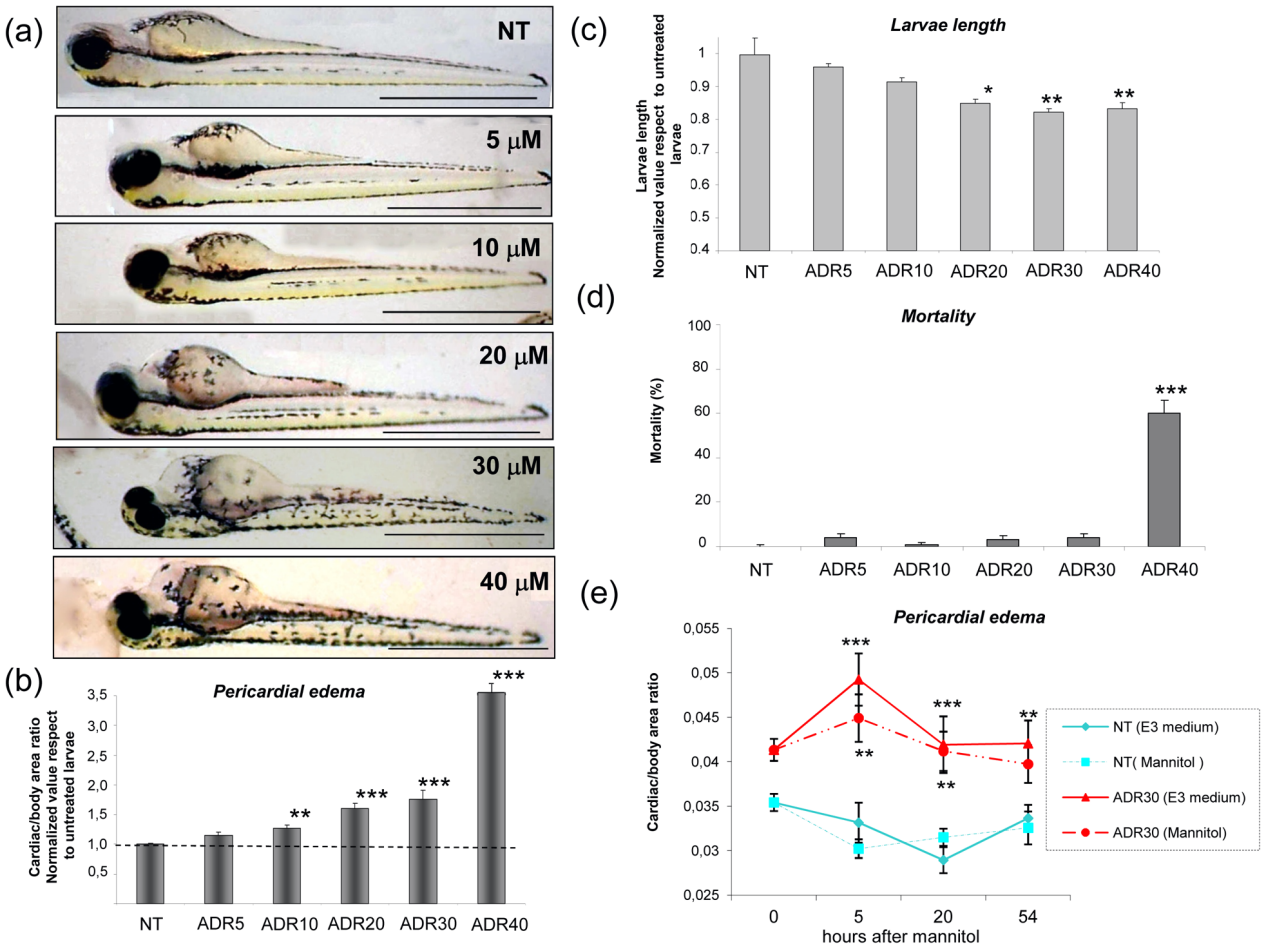
Dose-dependent effects of exposure to adriamycin at 9 hpf. (a) Lateral view of 72 hpf larvae exposed to medium (NT) or to adriamycin. Pericardial edema is evident at concentrations ranging from 10 µM to 40 µM. (b) The histogram displays cardiac area/total body area values, as an estimate of pericardial edema formation. Values are normalized to those of untreated larvae (NT). (c) At 72 hpf, larvae treated with adriamycin (ADR) 20–40 µM demonstrate a reduction of body length. (d) Mortality is significantly higher than controls at adriamycin concentration of 40 µM. (e) Temporal course of pericardial edema after addition of mannitol (175 mM) to the medium. The cardiac area/total body area remains significantly elevated in ADR-treated animals as compared to control (NT) larvae. Results are expressed as mean ±SEM of three independent experiments (15 fish for each experiment). Asterisks indicate significant differences between adriamycin-exposed and control (NT) animals: * = P<0.01; ** = P<0.005; *** = P<0.0001 vs NT. Scale bars  = 1 mm.

Consistently, at 72 hpf the cardiac/body ratio was increased as a consequence of any adriamycin dosage (5–40 µM), and a significant difference versus untreated embryos was reached by dosages from 10 to 40 µM (Figure 2a, 2b).

Treated embryos also showed a moderate reduction of length with dosages of 20–40 µM (Figure 2c). In addition, 40 µM adriamycin caused increased mortality among the animals (Figure 2d).

To assess if pericardial edema was due to glomerular damage or to alteration of other water barriers, such as gills and skin, we performed a mannitol test. Mannitol does not readily cross between biological compartments and its addition increases the osmolarity of water. Therefore, by abolishing the osmotic gradient between interior body fluids and water environment, mannitol is able to reduce pericardial edema when it is due to skin or gills failure [16]. As shown in Figure 2e, addition of mannitol to the medium did not reduce pericardial edema in adriamycin-treated embryos, therefore excluding the involvement of skin and gills, and supporting a possible alteration of kidney function.

**Figure 3:**
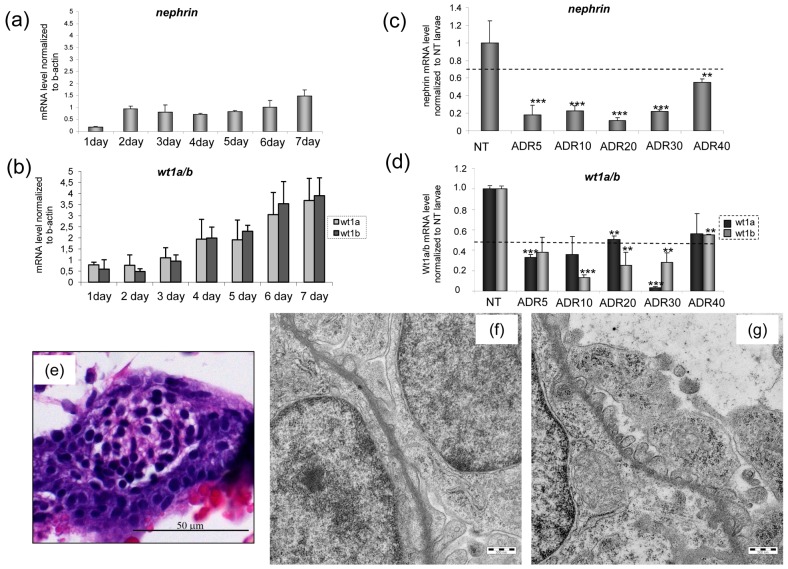
Temporal expression pattern of the podocyte markers nephrin (a) and wt1a/b (b) during early days of development and variation of expression after incubation with adriamycin (ADR) (c) (d). ** P<0.005 vs NT and *** P<0.0001 vs NT. Results are expressed as mean ±SEM of three independent experiments (10 fish per experiment). (e) At 72 hpf light microscopy of a treated fish does not show detectable damage. By transmission electron microscopy, impaired podocyte formation is evident in a treated (f) fish, with few and irregular podocyte processes. At the same time point, the untreated fish (g) shows regular distribution of processes along the glomerular basement membrane. Scale bars  = 500 nm.

To further prove glomerular involvement, we analyzed the expression of specific glomerular markers, namely wt1a, wt1b and nephrin. In keeping with previous results [18], [19], mRNA expression of nephrin, wt1a and wt1b in control embryos was already detectable at 1 dpf. A rapid increase of nephrin was observed at 2 dpf, then levels remained stable and showed a further mild increase at day 7 (Figure 3a). Wt1a and 1b expression progressively increased from day 3 to day 7 (Figure 3b).

Adriamycin-treated animals displayed a significant decrease of expression of glomerular markers at 72 hpf (Figure 3c,d), supporting podocyte damage as a cause of pericardial edema. Of note, the highest reduction of nephrin levels was obtained with adriamycin 20 µM.

The histological studies at 72 hpf control larvae showed completely formed glomeruli, but light microscopy was insufficient to reveal glomerular damage (Figure 3e), that was instead evident by transmission electron microscopy; TEM allowed detection of podocyte changes, consisting in the absence of process formation and covering of the basement membrane by flattened podocyte cytoplasm (Figure 3f). Control larvae presented instead regularly aligned primary and secondary processes along the basement membrane and initial appearance of slit diaphragms (Figure 3g).

As reported [8], after microinjection of 70-kDa FITC-labeled dextran control animals displayed heart fluorescence which progressively decreased from 1 h to 48 h. Eye fluorescence was stable up to 24 h, then declined at 48 h.

**Figure 4:**
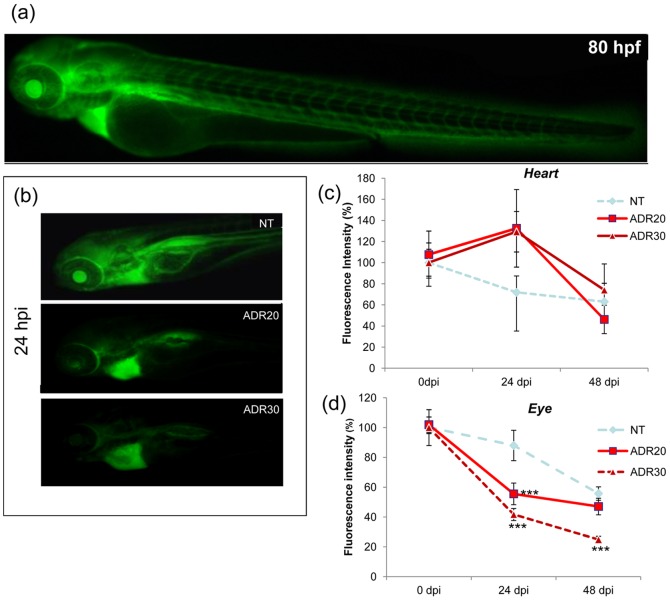
Clearance of 70-kDa dextran in 80 hpf larvae. (a) a control larva immediately after dextran injection (0 hpi) shows distribution of fluorescence through the body, with major fluorescence in the heart and the eye. (b) representative images of untreated and treated fish at 24 hpi. Exposure to adriamycin causes dose-dependent loss of eye fluorescence, whereas the marker is retained in the cardiac area, due to pericardial edema. The graphs show quantification data of fluorescence intensity (obtained from 30 fish per condition and time point) in the pericardial area (c) and the eye (d). *** = P<0.0001 vs NT.

Larvae exposed to 20 and 30 µM adriamycin had globally decreased fluorescence. FITC-labeled dextran accumulated in the pericardial area, due to edema (Figure 4b); consequently, heart fluorescence increased after 24 h and more slowly reached the value of untreated larvae, becoming comparable after 48 h from the injection (Figure 4b, c). Sustaining the hypothesis of a glomerular damage, with more rapid dextran clearance through the glomerulus, eye fluorescence declined rapidly and dose-dependently in fish exposed to adriamycin (Figure 4d).

### Persistence of glomerular damage after adriamycin removal

**Figure 5:**
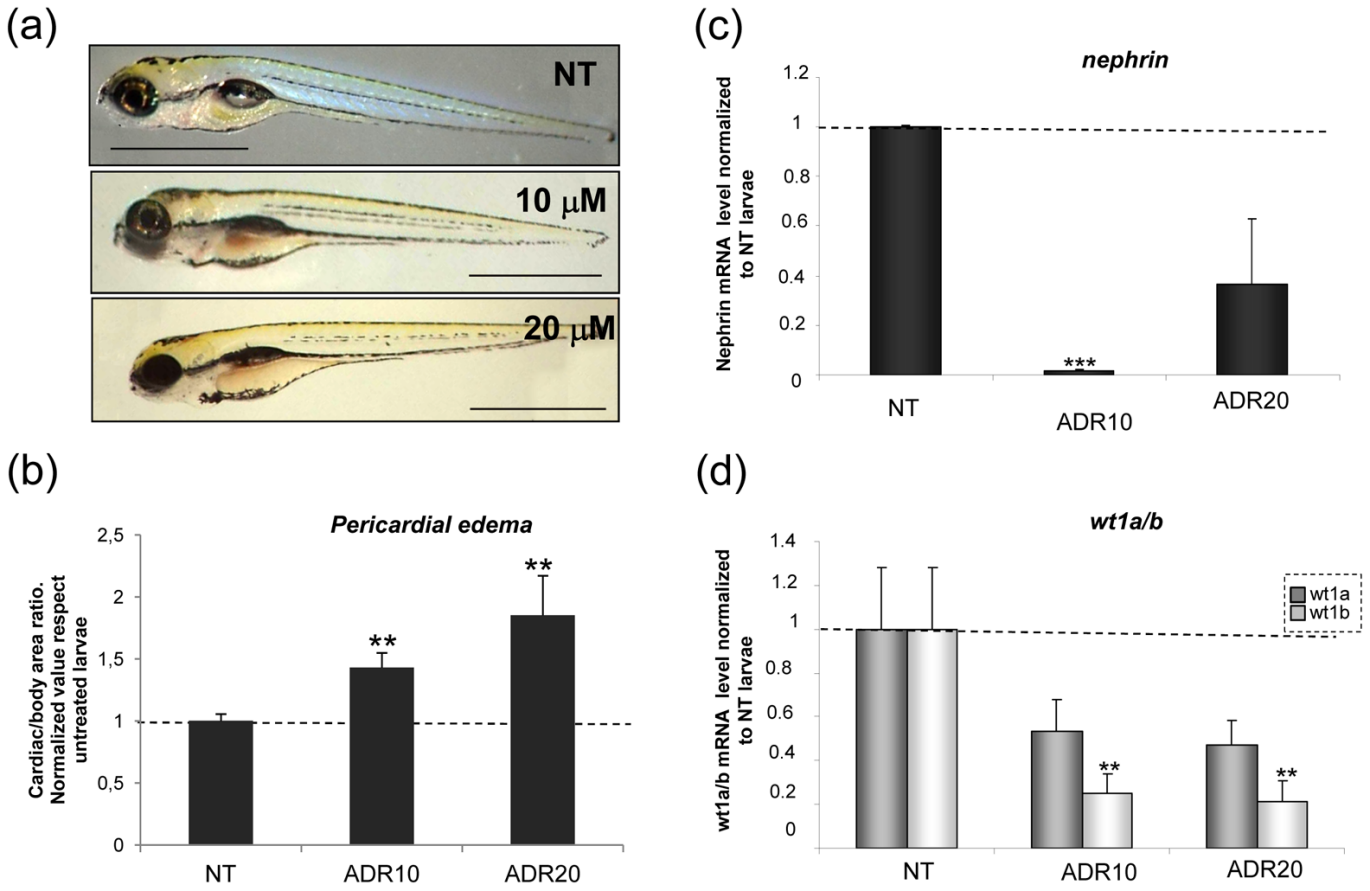
Analysis of adriamycin effects after removal from the medium. (a) At 120 hpf, treated larvae are characterized by persistence and worsening of pericardial edema. (b) The histogram displays values of cardiac area/total body area. (c) QRT-PCR analysis of podocyte markers. ** = P<0.005; *** = P<0.0001 vs NT.

Larvae exposed to adriamycin 10 and 20 µM were followed up to 120 hpf. In these larvae, pericardial edema progressively increased (Figure 5a, b) and was associated with further decline of the podocyte markers wt1a, wt1b, and nephrin (Figure 5c, d).

**Figure 6:**
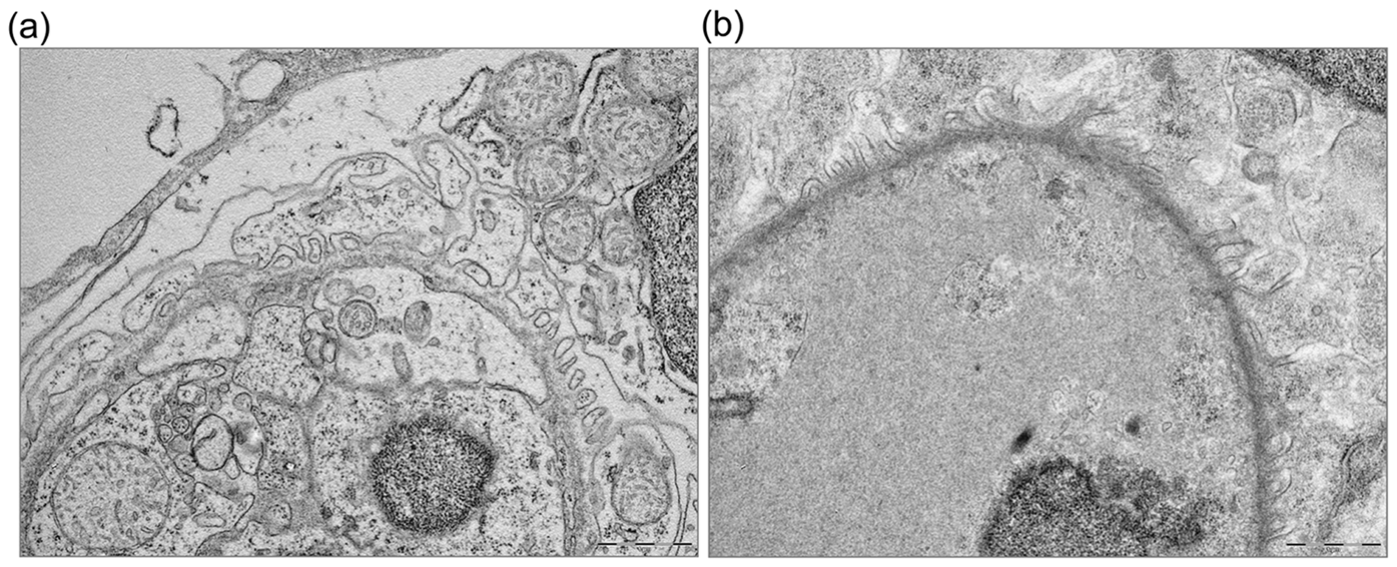
Ultrastructural analysis of glomeruli at 120 hpf. In NT fish the structure appears mature (a) while in adriamycin-treated fish there is almost complete disappearance of podocyte foot processes (b). Scale bars  = 1 µm.

In parallel severe podocyte damage was detected by TEM, with large areas of fusion, thinning, and microvillous degeneration of the foot processes, which were randomly attached to the glomerular basement membrane (Figure 6a), all features very different from normal mature podocytes observed at the same time point in control larvae (Figure 6b).

### Extra-renal effects of adriamycin: heart toxicity

Adriamycin is known to induce cardiomyopathy in humans [20], and to affect cardiac contractility in adult zebrafish [21]. Mechanistically, it has been hypothesized that cardiotoxicity is mediated by activation of free radicals-induced mitochondrial damage, which in turn imposes oxidative stress on the heart and triggers apoptotic cardiomyocyte death [22], [23]. In zebrafish embryos, cardiotoxicity has been reported with adriamycin concentrations higher than 40 µM [24] and manifests with loss of contractility and pericardial edema [25], [26].

Therefore, in our model it was essential to identify a dosage of adriamycin causing podocyte defects without cardiac side effects.

**Figure 7:**
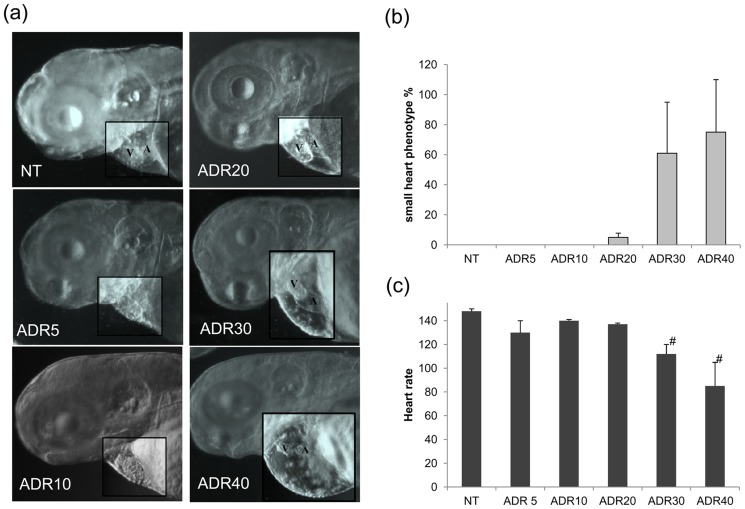
Heart morphology and functionality. (a) Morphological exam of the heart in the whole animal shows evident reduction of size and alteration of shape with adriamycin concentrations of 30 and 40 µM. (b) The graph shows the percentage of animals displaying heart changes. The high standard error proves the variability of the effect. (c) The heart rate shows a decrement in fish treated with 30–40 µM. ^#^ P<0.05 vs NT. A =  atrium V =  ventricle. Each data point is based on 30 embryos.

At 72 hpf the majority of animals given 30 (60% of larvae) and 40 µM (90% of larvae) adriamycin had a cardiac phenotype. Changes included heart malformation and decrease of the heart rate (Figure 7 a, b, c, Movie S3). Instead, embryos exposed to 10 and 20 µM adriamycin did not display changes of heart shape (Figure 7a) and the heart rate was not altered in this groups of animals (Figure 7b, c, Movie S1, Movie S2).

### Other extra-renal effects

To analyze other potential damaging effects of adriamycin in zebrafish embryos, we studied vessel, cartilage, and muscle formation.

Zebrafish angiogenesis starts at 24 hpf and is completed by 72 hpf. Subintestinal vessels (SIVs) are generally utilized in this type of studies because of their accessibility and relatively simple anatomy.

**Figure 8:**
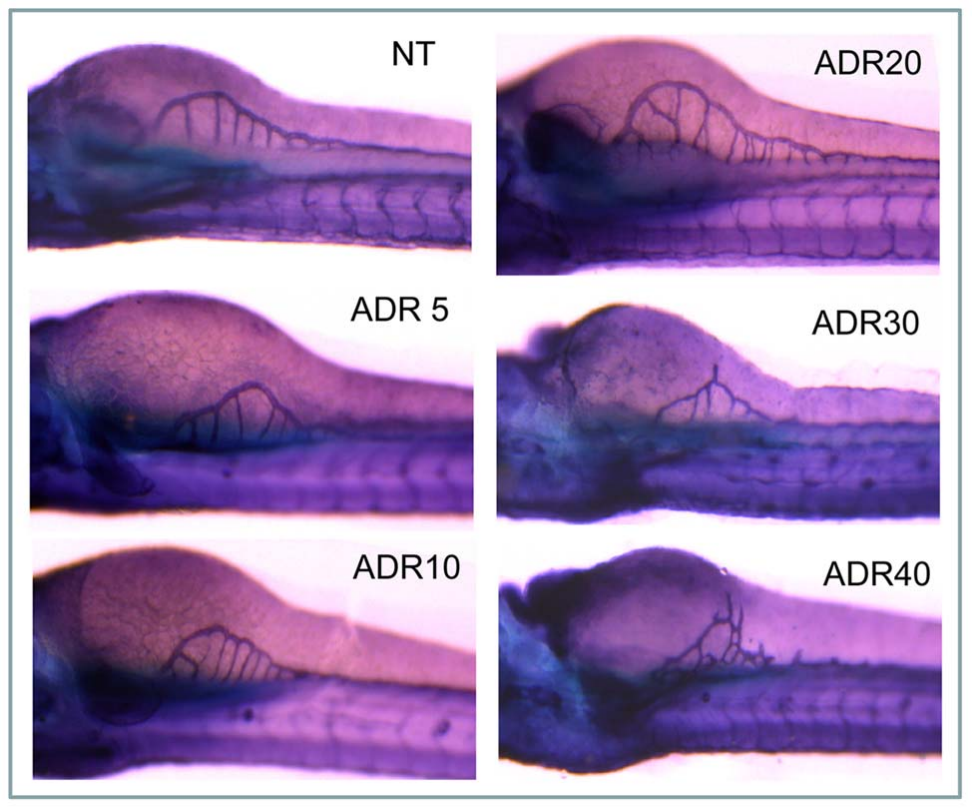
Subintestinal vessels. The Figureure shows subintestinal vessels (SIVs) observed at 72 hpf. Altered vessels are present in larvae treated with 30 and 40 µM of adriamycin.

We found normal SIV formation after 10 and 20 µM adriamycin, whereas at higher concentrations, particularly 40 µM, we observed the appearance of abnormal spikes projecting from the subintestinal vessel basket (Figure 8).

**Figure 9:**
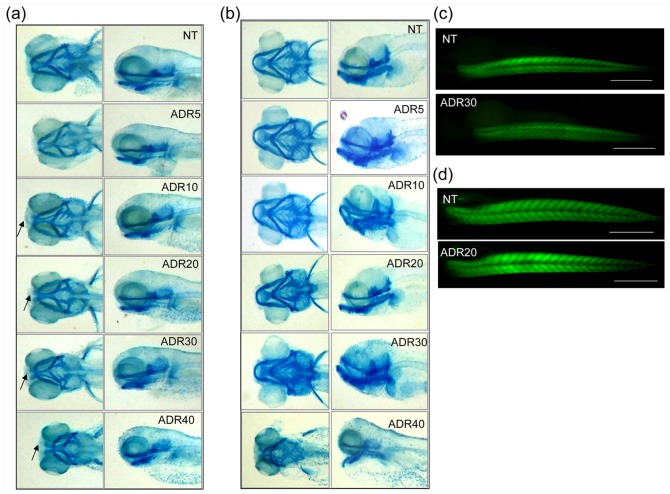
Cartilage development. At 72(a), the craniofacial cartilage, detected by alcian blue staining, shows a delay of development (arrows) in fish exposed to 10–40 µM adriamycin. (b) At 120 hpf, larvae exposed to adriamycin up to 30 µM are indistinguishable from the control in terms of craniofacial morphology. Instead, exposure to adriamycin 40 µM causes persistent alteration of the cartilage structure. FITC-phalloidin staining shows normal muscle morphology of 72 hpf (c) and 120 hpf (d) larvae exposed to 20 µM adriamycin.

In addition, adriamycin showed dose-dependent effects on the craniofacial cartilage, whose development was delayed (Figure 9a). Noteworthy, fish exposed to adriamycin 10 and 20 µM presented rapid recovery of cartilage development after adriamycin removal, as shown by alcian blue staining at 120 hpf (Figure 9b).

Instead, adriamycin did not influence muscle development, that was similar to controls for any adriamycin dosage during the whole observation period (Figure 9c,d).

## Discussion

Our results indicate that addition of adriamycin 10 and 20 µM to the medium of zebrafish embryos at 9 hpf, followed by its removal after 48 h, induces selective and progressive podocyte changes and a measurable alteration of permeability of the glomerular barrier.

Adriamycin is a cytotoxic anthracycline antibiotic used in antimitotic chemotherapy. In humans, it is infused intravenously to treat neoplastic diseases, such as acute leukemia, multiple myeloma, Hodgkin's and non-Hodgkin's lymphomas, and several sarcomas and carcinomas. The most common dosage is 60 to 75 mg/m^2^ of body surface. The drug has multiple side effects, such as nephrotoxicity, cardiotoxicity, and neurotoxicity.

Adriamycin is utilized by the nephrology research community primarily because it causes podocyte foot process effacement and nephrotic syndrome in rodents [11]. In cultured podocytes, addition of adriamycin determines rounding of the podocyte shape, with loss of actin stress fibers and loss of ramifications [27].

The primordial zebrafish pronephron appears at 19 hpf, during early somitogenesis, as a mass of intermediate mesoderm that lies under the second and third somite [28]. Therefore, by adding adriamycin at 9 hpf, when the embryo is in the gastrula period, the drug interferes with podocyte development, making our model useful to study a condition which resembles congenital nephrotic syndrome in mammals.

Among other molecules known to be harmful to podocytes, puromycin aminonucleoside has been applied to zebrafish embryos [8]; the authors microinjected 250–350 mg/kg puromycin aminonucleoside in 80 hpf larvae. At 80 hpf, the pronephron structure is already mature and active in its osmoregulatory function [29]; therefore the model can be used for studies addressing lesions of mature podocytes. Of note, to be effective in zebrafish, the required dosage of puromycin aminonucleoside is much higher than those required *in vitro* (10–60 µg/ml) and utilized in rodents (150 mg/kg).

Instead, our protocol applies the same dosages of adriamycin which are utilized on cultured podocytes [27]. Consistently, the same range of concentrations actively increase P_alb_ on isolated rat glomeruli.

Confirming data from the literature [25], even the lowest concentrations of adriamycin produced devastating effects if administered in the first 3 hours after fertilization, with death of all embryos by cardiac arrest, associated to head abnormalities and impaired eye formation.

While adriamycin toxicity has been mostly examined in adult zebrafish, there are not many studies on developmental toxicity. A recent publication [29] describes toxicity of adriamycin exposure lasting 96 h, with the substance dissolved in the medium at 72 hpf. The experiments show that toxicity effects, including heart rate changes, become highly significant as compared to control larvae for adriamycin dosages higher than 20 µM. Similar results were described by Huang et al. [24].

Albeit in these experiments adriamycin was added later and left in the medium longer than in our protocol, the data are concordant with our observations.

Adriamycin 30 and 40 µM administration caused relevant systemic effects in the majority of larvae. Instead, addition of 10–20 µM adriamycin determined almost exclusively glomerular effects and were characterized by a very low mortality rate in absence of major teratogenic effects. The main extraglomerular consequence we observed with these dosages was a delay of cartilage development, that rapidly reversed after elimination of the drug from the medium.

Instead, pericardial edema persisted and worsened after adriamycin removal, allowing further analysis of podocyte defective development. We performed different experiments to prove the prominent glomerular involvement caused by our experimental protocol, and results concordantly excluded the involvement of other water barriers and confirmed the altered morphology of podocytes, the increased clearance of fluorescent dextran, and the diminished expression of podocyte markers.

Wt1 and nephrin are essential regulators of nephrogenesis in zebrafish, particularly of glomerular structure formation. In contrast to other animals, fish possesses two wt1 paralogs, and during the first 24 hours of development they are expressed in an overlapping, though not identical, spatial and temporal pattern; after completion of pronephros differentiation, expression of wt1a and wt1b is limited to the podocytes [30], [31]. The crucial role of the wt1 in zebrafish pronephron development was shown in a study of RNA interference, in which the injection of wt1 morpholino arrested kidney development by inducing apoptosis of nephron progenitor cells [32]. Similarly, nephrin morpholino knockdown in zebrafish embryos caused formation of pericardial edema [33].

In our protocol, adriamycin decreased the expression of these genes, and expression levels remained lower than controls after removal from the medium.

In conclusion, we have established that low concentrations of adriamycin specifically target the pronephron structure, when dissolved in the medium at 9 hpf. Main advantages of this model are the feasibility and high reproducibility, making it prospectively useful to analyze podocyte development and to test drug efficacy in a very severe condition of podocyte developmental damage.

## Supporting Information

Movie S1 **Recording of heart beating in untreated larvae at 72 hpf.**
(ZIP)

Movie S2 **Recording of heart beating in larvae exposed to 20 µM adriamycin at 72 hpf.** There are no appreciable differences as compared to untreated larvae.
(ZIP)

Movie S3 **Recording of heart beating in larvae exposed to 40 µM adriamycin at 72 hpf.** Distortion of the heart shape and change of contractility are clearly detectable.
(ZIP)
