# Correction: Podocyte Developmental Defects Caused by Adriamycin in Zebrafish Embryos and Larvae: A Novel Model of Glomerular Damage

**DOI:** 10.1371/journal.pone.0105848

**Published:** 2014-08-12

**Authors:** The PLOS ONE Staff

There is an error in affiliation 3 as it should be divided into two separate institutions. Massimo Mariotti is affiliated with:

3 Department of Biomedical, Surgical and Dental Sciences, University of Milan, Milan, Italy

8 IRCCS Galeazzi Orthopedic Institute, Milan, Italy
